# A Small Auxin-Up RNA Gene, *IbSAUR36*, Regulates Adventitious Root Development in Transgenic Sweet Potato

**DOI:** 10.3390/genes15060760

**Published:** 2024-06-10

**Authors:** Yuanyuan Zhou, Aixian Li, Taifeng Du, Zhen Qin, Liming Zhang, Qingmei Wang, Zongyun Li, Fuyun Hou

**Affiliations:** 1 Crop Research Institute, Shandong Academy of Agricultural Sciences, Jinan 250100, China; 2 Key Laboratory of Phylogeny and Comparative Genomics of the Jiangsu Province, School of Life Sciences, Jiangsu Normal University, Xuzhou 221116, China

**Keywords:** *IbSAUR36*, adventitious root development, lignin, IAA, JA, sweet potato

## Abstract

Small auxin-upregulated RNAs (*SAURs*), as the largest family of early auxin-responsive genes, play important roles in plant growth and development processes, such as auxin signaling and transport, hypocotyl development, and tolerance to environmental stresses. However, the functions of few *SAUR* genes are known in the root development of sweet potatoes. In this study, an *IbSAUR36* gene was cloned and functionally analyzed. The IbSAUR36 protein was localized to the nucleus and plasma membrane. The transcriptional level of this gene was significantly higher in the pencil root and leaf.This gene was strongly induced by indole-3-acetic acid (IAA), but it was downregulated under methyl-jasmonate(MeJA) treatment. The promoter of *IbSAUR36* contained the core *cis*-elements for phytohormone responsiveness. Promoter β-glucuronidase (GUS) analysis in *Arabidopsis* showed that *IbSAUR36* is highly expressed in the young tissues of plants, such as young leaves, roots, and buds. *IbSAUR36*-overexpressing sweet potato roots were obtained by an efficient *Agrobacterium rhizogenes*-mediated root transgenic system. We demonstrated that overexpression of *IbSAUR36* promoted the accumulation of IAA, upregulated the genes encoding IAA synthesis and its signaling pathways, and downregulated the genes encoding lignin synthesis and JA signaling pathways. Taken together, these results show that *IbSAUR36* plays an important role in adventitious root (AR) development by regulating IAA signaling, lignin synthesis, and JA signaling pathways in transgenic sweet potatoes.

## 1. Introduction

Sweet potato(*Ipomoea batatas* (L.) Lam.) is an important tuberous root crop cultivated worldwide [1,2,3]. The tuberous root development is a complex biological process for sweet potato yield [4,5,6]. The sweet potato adventitious roots (ARs) originated from root primordia located on the nodes as well as at the cut ends. These ARs became ‘thick’ pigmented storage roots(SRs), ‘thick’ pigmented pencil roots (PRs), and white fibrous roots (FRs) [7,8]. Therefore, they can contribute to improving sweet potato yield and development.

In general, the root expansion is not only regulated by endogenous phytohormones and genes, but is also affected by the external environment [9,10,11]. Previous studies have demonstrated that auxins, mainly indole-3-acetic acid (IAA), play an essential role in the initiation of SR swelling, and auxin concentration increase with the increase inroot diameter in the early stage of root expansion [12,13]. During the early stage of storage root development, the endogenous IAA content and SRD1 transcript level increased concomitantly, *SRD1*-overexpressing transgenic sweet potato plants cultured in vitro produced thicker and shorter fibrous roots than wild-type plants, suggesting an involvement of *SRD1* during the early stage of the auxin-dependent development of the storage root [14]. The early accumulation of IAA in the rooting zone stimulated the formation of ARs in cuttings [13,15]. Many studies suggest that the genes related to phytohormone, IAA, jasmonic acid (JA), and lignin biosynthesis are widely used for clonal plant propagation in sweet potato SRs [15,16,17].

Plants can quickly sense and respond to changes in auxin levels, and these responses involve several major classes of auxin-responsive genes, including the auxin/indole-3-acetic acid (*Aux*/*IAA*) family, the auxin response factor (*ARF*) family, small auxin-upregulated RNAs (*SAURs*), and the auxin-responsive Gretchen Hagen3 (*GH3*) family [18].

*SAURs* are early auxin-responsive genes, with a large family in plants [19,20,21,22]. Many studies suggest that the *SAURs* gene family plays important roles in root formation. The overexpressionof *CsSAUR31* exhibited longer roots in cucumber [23]. It revealed that *OsSAUR11* positively enhanced the ratio of deep rooting in transgenic rice [24]. The PagWOX11/12a-PagSAUR36 module significantly promoted AR development via the auxinsignaling pathway in transgenic poplar [25]. However, it is unclear whether some *SAURs* participate in the development of ARs in sweet potato.

The initiation of SR bulking and the subsequent thickening process of sweet potatoes is a complex biological process, including the accumulation of morphogenesis and assimilation products. Such information does not reflect an accurate relationship between those ARs andstorage root development, and it is unclear how many of those ARs actually become SRs. Also, it is unclear whether SRs emanate from these initial ARs. Elucidating the molecular mechanisms of AR development can contribute to the root architecture. Here, we identified a candidate *SAUR* gene, *IbSAUR36*, which regulates AR development in sweet potatoes. The results suggest that *IbSAUR36* may be a useful potential target for further molecular breeding of high-yielding sweet potatoes.

## 2. Materials and Methods

### 2.1. Plant Materials

Sweet potato cultivars Jishu25 (JS25), Jishu29 (JS29), and Xuzihsu 8(XZ8) were planted in the greenhouse of the Crops Research Institute, Shandong Academy of Agricultural Sciences, Jinan, China. JS25 and JS29 were used to isolate the *IbSAUR36* gene and analyze the expression in different tissues, respectively. XZ8 has been used to characterize the gene functionas described by Yu et al. (2020) [26]. The function of the *IbSAUR36* promoter was identified using *Arabidopsis thaliana* (Columbia-0, wild type, WT).

### 2.2. Cloning and Sequence Analysis of IbSAUR36 and Its Promoter

The Trizol Up Kit (ET111, Transgen, Beijing, China) was used to extract the total RNA and then transcribe the first-strand cDNA with the PrimeScript^TM^ RT reagent kit and gDNA Eraser kit (PR047A, Takara, Beijing, China). The full-length cDNA of *IbSAUR36* was amplified from the first-strand DNA using the homologous cloning method with specific primers. The open-reading frame (ORF) of *IbSAUR36* was analyzed with ORF Finder. The molecular weight and theoretical isoelectric point (*p*I) of IbSAUR36 were calculated with ProtParam tool (https://web.expasy.org/protparam/, accessed on 1 December 2023). Amino acid sequence alignment was analyzed using DNAMAN V6 software. The phylogenetic tree was constructed with MEGA 7.0 software with 1000 bootstrap replicates. Genomic DNA was extracted using the cetyltrimethylammonium bromide (CTAB) method. The genomic sequence and promoter sequence of *IbASUR36* were amplified from genomic DNA using the homologous cloning method with specific primers. All the specific primers are shown in Supplementary Table S1. The *cis*-acting regulatory elements in its promoter were analyzed using PlantCARE (http://bioinformatics.psb.ugent.be/webtools/plantcare/html/, accessed on 1 December 2023).

### 2.3. Expression Analysis

The transcript levels of *IbSAUR36* in leaf, stem, hair root, pencil root and storage root tissues of the 125-day-old field-grown plants of JS25 and JS29 was analyzed with quantitative real-time PCR (qRT-PCR). Furthermore, the 2-week-oldof JS25 and JS29 plants were stressed in Hoagland solutionwith H_2_O (control) and 100 mM IAA, and 100 mM MeJA, respectively, and sampled at 0, 1, 3, 6, 12 and 24 h after treatments for analyzing the expression of *IbSAUR36. Ibactin* (AY905538) was used to normalize the expression levels in sweet potato. All the specific primers are showed in Supplementary Table S1.

### 2.4. Function Analyse of IbSAUR36 Promoter

The promoter of *IbSAUR36* was inserted into the DX2181 vector with the β-glucuronidase (*GUS*) gene, and then it was transferred into the *Agrobacterium tumefaciens* strain GV3101. The transgenic *Arabidopsis* plants were produced and further grown in pots to obtain T_3_ seeds. The GUS activity in transgenic *Arabidopsis* was examined by GUS histochemical staining solution. After staining, tissues were cleared by replacing the staining solution with several changes of 70% (*v/v*) and 90% (*v/v*) ethanol as necessary.

### 2.5. Regeneration of the Transgenic Sweet Potato Plants

The coding region of *IbSAUR36* was inserted into a pUBI.U4::*IbSAUR36*-CaMV35S::DsRed expression cassette pNRT expression vector. The constructs wereused for *A*. *rhizogenes*-mediated transformation as described by Yu et al. (2020) [26]. The transgenic roots were harvested with the help of red fluorescent protein (RFP). In order to explore the cross-sectional structural characteristics of the root cells, the root tissues were collected from CK and transgenic lines for paraffin sections and dyed with sarranine and solid green. Theexpression level of *IbSAUR36* was analyzed with qRT-PCR using primers in different roots (Supplementary Table S1). The phytohormone contents of the roots were measured using liquid chromatography tandem mass spectrometry (LC-MS/MS).

### 2.6. RNA-Sequencing

To analyze the function of *IbSAUR36*, the roots of transgenic sweet potato and CK were used for RNA-sequencing (RNA-seq). The RNA-seq library was constructed using an Ultra RNA sample preparation kit (Illumina, San Diego, CA, USA). Fragments were sequenced using an Illumina HiSeq 2500 according to the standard method (Illumina). Total reads were mapped to the *Ipomoeatrifida* genome (sweet potato GARDEN (kazusa.or.jp)). Differentially expressed genes were identified using Cuffdiff with default criteria (fold change > 1.5) and an adjusted false discovery rate (*p* value < 0.05). Three independent biological replicates were used for the RNA-sequencing analysis. An analysis using the Kyoto Encyclopedia of Genes and Genomes (KEGG) pathway was conducted according to database instructions (KEGG PATHWAY Database). The gene expression patterns were graphically represented in a heat map by cluster analysis using TBtools software v2.041.

### 2.7. Statistical Analysis

Three biological replicates were conducted and the data were presented as the mean ± SE. All were analyzed using Student’s *t*-test (two-tailed analysis). Significance levels at *p* < 0.05 weredenoted bydifferent small letters, respectively.

## 3. Results

### 3.1. Identification of IbSAUR36 and Its Promoter in Sweet Potato

**Figure 1:**
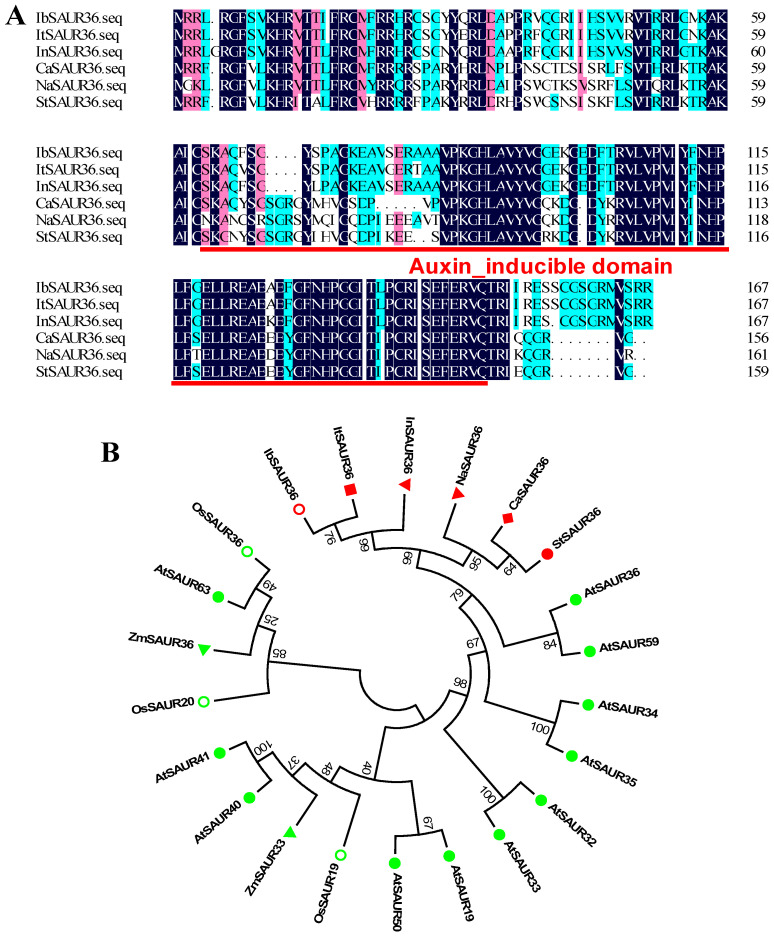
Multiple alignment (**A**) and phylogenetic analysis (**B**) of IbSAUR36with its homologs from other plants.

We obtained a SAUR family gene from the previous comparative transcriptome analysis of root development in two sweet potato cultivars, JS25 and JS29. This gene was named *IbSAUR36* by homology analysis. The 626-bp cDNA sequence of *IbSAUR36* included 507-bp ORF and encoded a protein of 168 aa (molecular weight: 19.167 kDa) with a predicted *p*I of 10.98. Multiple sequence alignment showed that the new SAUR protein contained an auxin-inducible domain and has high homology with SAUR36 proteins from *Ipomoea triloba* (ItSAUR36, XP_031111161.1, 96.4%), *Ipomoea nil* (InSAUR36, XP_019189087.1, 93.5%), *Capsicum annuum* (CaSAUR36, XP_016557650.2, 60.1%), *Nicotiana attenuata* (NaSAUR36, XP_019258606.1, 57.6%), and *Solanum tuberosum* (StSAUR36, XP_006347888.1, 56.4%) in the NCBI database (Figure 1A). Phylogenetic analysis of SAUR proteins with a neighbor-joining method revealed that the new SAUR protein has high homology with ItSAUR36 proteins from *Ipomoea triloba* (Figure 1B). The SAUR gene sequence is the same as that of IbSAUR36 (Ibat.Brg.04E_G002670.1) from the updated genome assembly and annotation for the sweet potato variety *I. batatas* Beauregard. Thus, the new *SAUR* gene was named *IbSAUR36*. The 1494-bp promoter region of *IbSAUR36* contained several phytohormone-responsive, *cis*-acting regulatory elements (Supplementary Figure S1).

### 3.2. Expression Analysis

**Figure 2:**
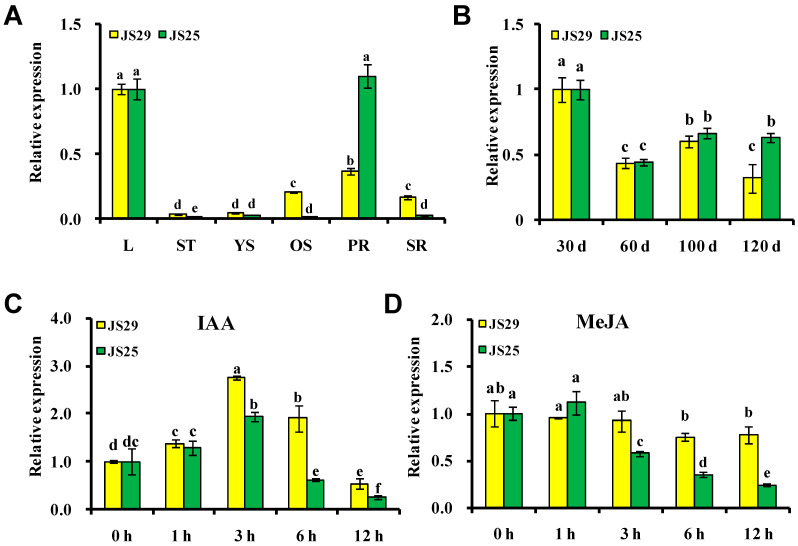
Relative expression of *IbSAUR36*. (**A**) Relative expression of *IbSAUR36* in different tissues of 125-day-old field-grown JS25 and JS29. L, leaf; ST, stem tip; YS, young stem; OS, old stem; PR, pencil root; SR, storage root. (**B**) Relative expression of *IbSAUR36* in different developmental stages of JS25 and JS29. d, day. (**C**,**D**) Relative expression of *IbSAUR36* in JS25 and JS29 after IAA or MeJA treatments. h, hour.The different small letters indicate a significant difference at *p* < 0.05 according to Student’s *t*-test.

To study the potential function of *IbSAUR36* in sweet potato, the expression was analyzed with qRT-PCR in different tissues and treatments of JS25 and JS29. For the field-grown plants, the expression level of *IbSAUR36* was highest in the leaves and pencil roots (Figure 2A). The *IbSAUR36* was downregulated in different developmental stages of the storage roots after 30 days (Figure 2B). The expression of *IbSAUR36* peaked at 3 h after IAA treatment (Figure 2C) and downregulated after MeJA treatment (Figure 2D). These results suggest that *IbSAUR36* might be involved in root development, IAA, and JA response pathways.

### 3.3. Functional Analysis of IbSAUR36 Promoter

**Figure 3:**
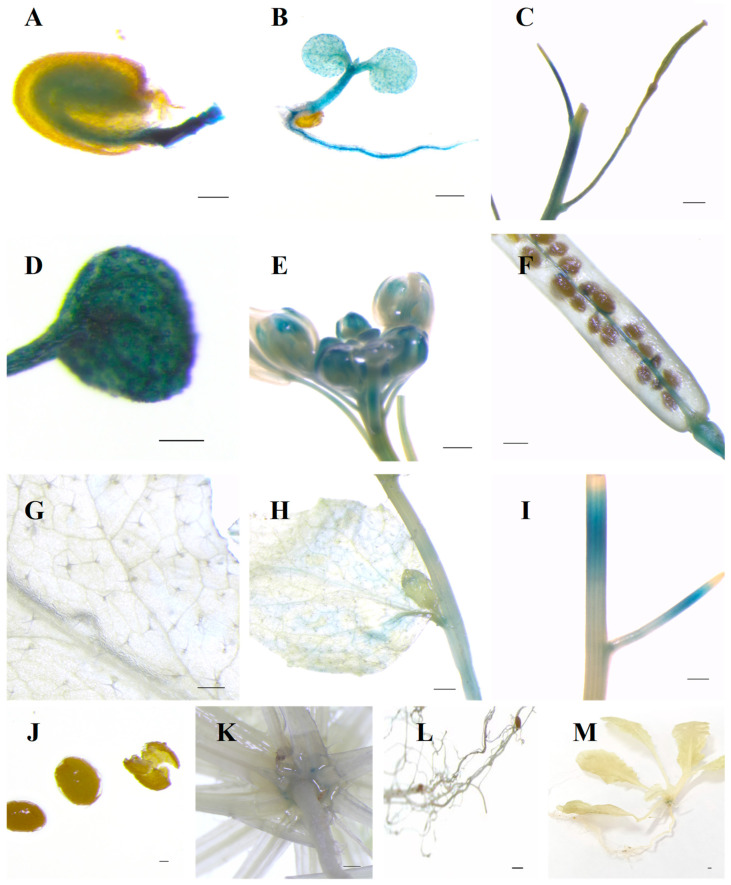
Tissue-specific localization of the IbSAUR36 protein in transgenic *Arabidopsis* identified by histochemical analysis of GUS activity driven by the *IbSAUR36* promoter. (**A**) The germinating seed. (**B**) 4-day-old seedling. (**C**) Immature silique. (**D**) Leaf in immature stage. (**E**) Bud. (**F**) Silique in mature stage. (**G**) Rosette leaf. (**H**) Internode with leaf. (**I**) Internode. (**J**) Seed. (**K**) Rosette. (**L**) Mature root. (**M**) Col-0. Bars = 100 μm (**A**,**D**,**J**,**L**), 500 μm (**B**,**C**,**E**–**I**,**K**,**M**).

The promoter region of *IbSAUR36* contained several phytohormone-responsive *cis*-acting regulatory elements, including auxin, MeJA, SA, zein and ABA (Figure S1). The *IbSAUR36* promoter fused to the *GUS* reporter gene has been introduced into *Arabidopsis*. GUS analysis of different tissues showed that *IbSAUR36* was active in the young tissues of plants, such as the germinated seed, immature plant root, fruit, and bud of transgenic *Arabidopsis* (Figure 3). These results suggest that *IbSAUR36* might be involved in tissue development through plant hormone pathways.

### 3.4. IbSAUR36 Affected Phytohormone Homeostasis in Transgenic Sweet Potato Roots

**Figure 4:**
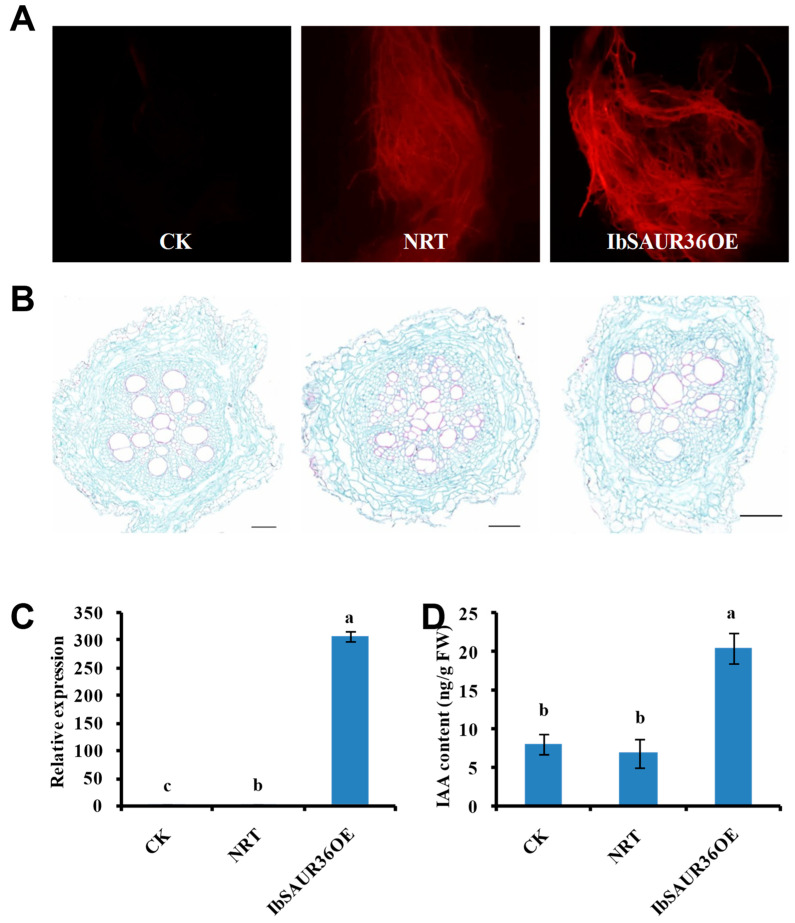
Production of the *IbSAUR36*-overexpressing sweet potato roots. (**A**) The root of transgenic sweet potato under red fluorescent protein(RFP) laser irradiation. (**B**) The histological analysis of transgenic sweet potato roots and CK. Bar = 0.1 mm. (**C**) Expression analysis of *IbSAUR36* in the transgenic roots and CK. (**D**) IAA content of transgenic sweet potato roots and CK. CK, roots of XZ8. NRT, roots of pNRT transgenic sweet potato. IbSAUR36OE, roots ofpNRT-*IbSAUR36* transgenic sweet potato. The data are presented as the means ± SEs (n = 3). The different small letters indicate a significant difference at *p* < 0.05 according to Student’s *t*-test.

To investigate the role of the *IbSAUR36* gene in phytohormone signaling pathways of sweet potato, the overexpression vector pNRT-*IbSAUR36* was constructed and introduced into sweet potato XZ8, while the XZ8 (CK) and the XZ8 introduced by pNRT (NRT) served as as negative controls. The RFP signal was observed in transgenic roots, but not in CK roots (Figure 4A). As shown in Figure 4B, the histological analysis of the transverse section revealed a higher number of lignified cells around xylem bundles in NRT roots than that of the CK lines. However, there was no obvious change in lignin deposition patterns in the roots between the IbSAUR36OE and CK lines. A qRT-PCR analysis revealed that the expression level of *IbSAUR36* was significantly increased in *IbSAUR36-*overexpressingroots compared with that of CK and NRT roots (Figure 4C). Meanwhile, the IAA content significantly increased in *IbSAUR36-*overexpressing roots (Figure 4D).

### 3.5. IbSAUR36 Regulates the Genes Involved in IAA, JA, and Lignin Signaling Pathways

**Figure 5:**
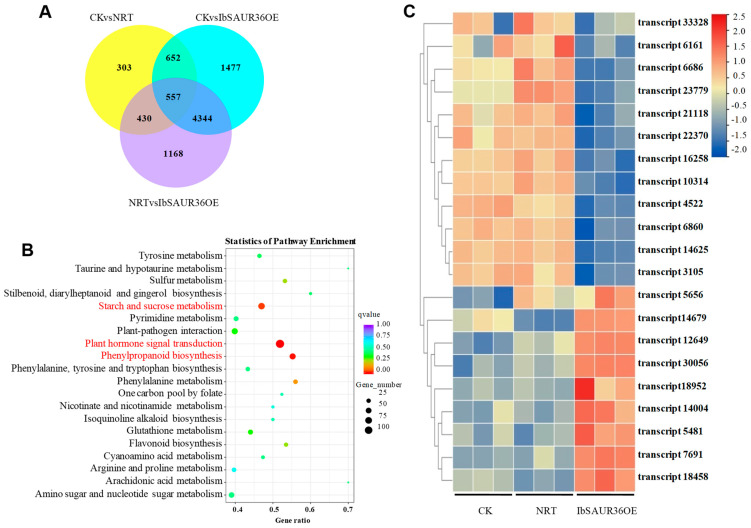
Venn, KEGG, andexpression analyses of DEG in transgenic sweet potato. (**A**) Venn analysis. (**B**) KEGG analysis. (**C**) Expression analysis.

To explore the mechanism of *IbSAUR36* in the transgenic roots, differentially expressed genes and metabolic pathways in transgenic sweet potato were analyzed by RNA-Seq. Using CK and NRT as the control groups, we obtained a total of 8931 differentially expressed genes (DEGs), of which, 4344 genes were further analyzed by the KEGG database (Figure 5A). KEGG enrichment analysis showed that the 4344 DEGs in overexpressing roots were primarily enriched plant phytohormone signal transduction, phenylpropanoid biosynthesis, and starch and sucrose metabolism pathways (Figure 5B).

The DEGs of auxin biosynthesis (transcript14004, transcript18952) and import transduction (transcript7691) pathway were upregulated, while the auxin transferred gene (transcript14625), auxin outport gene (transcript23779), auxin signal transduction genes (transcript3105, transcript21118), JA-associated genes (transcript6686, transcript6860, transcript4522 and transcript6161), and lignin biosynthesis genes (transcript10314, transcript22370) were downregulated (Figure 5C). The results indicate that overexpression of *IbSAUR36* might regulate the IAA, JA, and lignin pathways.

### 3.6. IbSAUR36 Regulated the Genes Involved in Root Development

**Figure 6:**
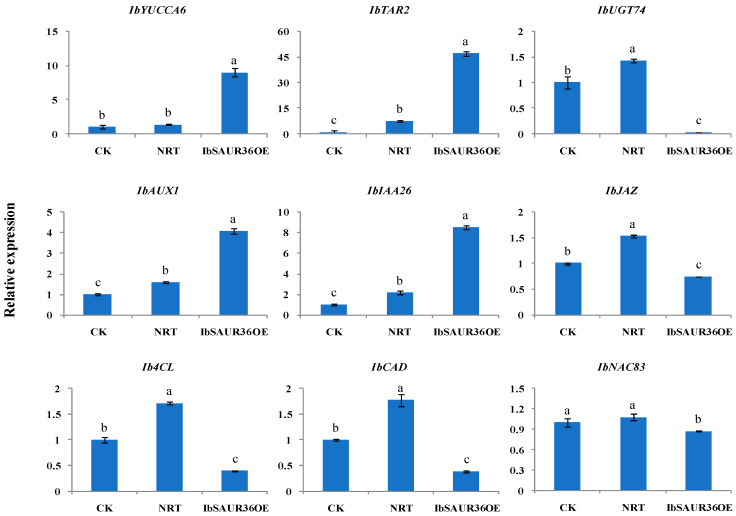
Expression of the genes related to root development in *IbSAUR36* overexpressing lines. CK, root of XZS8. NRT, root of pNRT transgenic sweet potato. IbSAUR36OE, root of pNRT-*IbSAUR36* transgenic sweet potato. The data are presented as the means ± SEs (n = 3). The different small letters indicate a significant difference at *p* < 0.05 according to Student’s *t*-test.

The initiation and development of SRs are intricately regulated by a transcriptional regulatory network, including the biosynthesis of lignin, flavanols, and starch. To explore the mechanism of *IbSAUR36* in the pre-swelling stage, we chose 9 storage-associated genes and analyzed their relative expression. The results showed that auxin pathway enzyme genes *IbYUCCA6*, *IbTAR2*, *IbAUX1*, and *IbIAA26* were upregulated, while the auxin glycosyltransferase gene *IbUGT74*, JA signal transduction enzyme gene *IbJAZ*, lignin biosynthesis enzyme genes *Ib4CL* and *IbCAD*, and root development-related transcription factor gene *IbNAC83* were downregulated in *IbSAUR36* overexpressing roots (Figure 6).

## 4. Discussion

### 4.1. IbSAUR36 Is anImportant Factor in Auxin and JA Signaling Pathways

The phytohormone auxin and JA play essential regulatory roles in multiple aspects of plant growth and development and in stress responses [27,28,29]. The YUCCA and TAR members are key enzymes of the IAA-biosynthetic pathway [30,31]. Glycosylation of IAA is carried out by UDP-glycosyltransferase 84B1 (UGT84B1), while UGT74D1 is implicated in the glycosylation of the irreversibly formed IAA catabolite oxIAA in *Arabidopsis* [32]. The content of IAA and expression levels of *IbYUCCA6* and *IbTAR2* were significantly increased, while those of *IbUGT74* were significantly reducedin the *IbSAUR36*-overexpressing sweet potato roots (Figure 5 and Figure 6). Further, the auxin-responsive genes, including *Aux/IAA*, *ARF*, *SAUR*, and *GH3*, have roles in the tuberization process in sweet potatoes [33]. Many studies have shown that the genes in the auxin signaling pathway are able to regulate the genes in the JA pathway. *StARF16* regulates defense gene *StNPR1* during the JA-mediated defense response upon necrotrophic pathogen interaction [34]. The GH3 genes are cytosolic, acidic amido synthetases of the firefly luciferase group that conjugate auxins, jasmonates, and benzoate derivatives to a wide group of amino acids [35]. *SAURs* and *GH3* have been shown to be specifically induced by the plant hormone auxin and JA. The transcription factors MYC2 and SAUR21 play a central role in the hormonal balance between JA-Ile and IAA [36]. The JA receptor JASMONATE-ZIM DOMAIN (JAZ) protein, JAZ4, has a prominent function in canonical JA signaling as well as the auxin signaling pathway [37]. The *SAUR* family is an important factor in auxin signal transduction pathways, which include approximately 200 members in sweet potato [33]. In this study, we characterized an auxin-regulated gene, *IbSAUR36*, in sweet potato. This *IbSAUR36* gene was upregulated after IAA treatment and downregulated after JA treatment (Figure 2). The *IbSAUR36*-overexpressing sweet potato root exhibited a better RFP signal and more accumulation of IAA (Figure 5). The genes in auxin and JA signaling pathways were upregulated in the *IbSAUR36*-overexpressing sweet potato roots (Figure 6). Based on all the above results, we propose that *IbSAUR36* positively regulates the IAA and the JA signaling pathways.

### 4.2. IbSAUR36 Regulated the Lignin in Root Development

Numerous studies have confirmed that auxin plays a key role in AR formation [13,38,39,40,41]. *SAUR* is the largest gene family with auxin-responsive factors [21]. The main candidate genes, including *MdSAUR2*, *MdSAUR29*, *MdSAUR60*, *MdSAUR62*, *MdSAUR69*, *MdSAUR71*, and *MdSAUR84*, regulated the root growth angle in apples [42]. It was found that *PbrSAUR13* promoted the synthesis and accumulation of stone cells and lignin, while *PbrSAUR52* inhibited the synthesis and accumulation of stone cells and lignin [43]. *OsSAUR11* positively regulates deep rooting in rice through participating in the regulation of auxin signaling [24]. The findings revealed high levels of *CsSAUR31* expression within the root and male flower tissues, and *CsSAUR31-*overexpressing plants exhibited longer roots and hypocotyls in cucumbers [23]. However, the function of the *SAUR* gene family in sweet potato is largely obscure.

Sweet potato is an important tuberous root crop, and its yield depends on a change in the developmental fate of ARs into SRs [2,44]. The lignin biosynthesis limits sweet potato yield formation in storage roots [45]. The AP2/ERF transcription factor, IbRAP2.4, inhibited SR formation in transgenic sweet potato by comprehensively upregulating lignin biosynthesis pathway genes [46]. Transcriptional profiling of sweet potato roots indicates down-regulation of lignin biosynthesis and up-regulation of starch biosynthesis at an early stage of SR formation [47]. The lignin biosynthesis genes *IbPAL*, *IbC4H*, *Ib4CL*, *IbCCoAOMT*, and *IbCAD* were upregulated under GA treatment in the sweet potato roots [5]. *MtCAD1* knockout medicago truncatula plants have reduced lignin content and display slower growth than WT [48]. *IbCAD1-*overexpressing plants displayed lower root weights and lower ratios of tuberous roots to pencil roots than WT [49]. In this study, overexpression of *IbSAUR36* regulatedlignified cell development in ARs (Figure 4B). Further, the lignin biosynthesis genes *Ib4CL* and *IbCAD* were downregulated in the *IbSAUR36*-overexpressing sweet potato roots (Figure 6). The results show that *IbSAUR36* may regulate the lignin biosynthesis in root pre-swelling development.

## 5. Conclusions

In this study, an *IbSAUR36* gene was cloned and functionally analyzed. We demonstrated that the overexpression of *IbSAUR36* promoted the accumulation of IAA, upregulated the genes encoding IAA synthesis and its signaling pathways, and downregulated the genes encoding lignin synthesis and JA signaling pathways. Taken together, these results show that overexpression of *IbSAUR36* may impact root development by IAA signaling, lignin synthesis, and JA signaling pathways in transgenic sweet potato.

## Data Availability

The original contributions presented in the study are included in the article/supplementary material, further inquiries can be directed to the corresponding author/s.

## Supplementary Materials

The following supporting information can be downloaded at: https://www.mdpi.com/article/10.3390/genes15060760/s1, Figure S1: Promoter of *IbSAUR36* showing different *cis*-acting regulatory elements associated with phytohormone responses. Table S1: Primers used in this study.
