# Supplementary material for: A Small Auxin-Up RNA Gene, IbSAUR36, Regulates Adventitious Root Development in Transgenic Sweet Potato
Source: Genes (Basel). 2024 Jun 10;15(6):760. doi: 10.3390/genes15060760 (PMC11203243; doi:10.3390/genes15060760)
Supplement: Supplementary file 1 [file genes-15-00760-s001.zip › genes-3007792-supplementary.pdf]

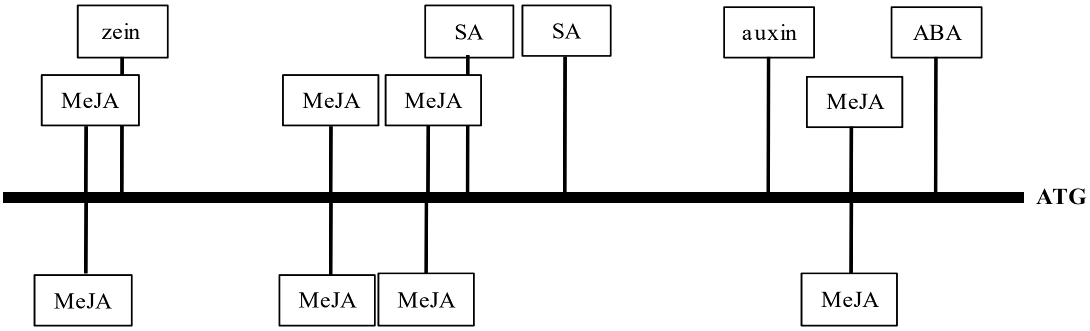

Figure S1. Promoter of *IbSAUR36* showing different cis-acting regulatory elements associated with phytohormone responses.

Table S1. Primers used in this study.

Primer name	Primer sequence (5'-3')
Primers for 5'-promoter region	
IbSAUR36-Pro-F	CCACAAACTGTCCTT
IbSAUR36-Pro-R	ACTCGCAGGAAACCCA
Primers for vector construction	
IbSAUR36-cDNA-F	CTGTTGTTTGCGAATATGCG
IbSAUR36- cDNA-R	CCAACAAGCTAATAGTGGGCCA
IbSAUR36- DX2181-F-(*Hind* III)	GATCTACAGCGCTGAAGCTTCCACAAACTGTCCTT
IbSAUR36- DX2181-R-(*Bam* HI)	GACTGACCACCCGGGGATCCACTCGCAGGAAACCCA
Ib SAUR36-pNRT-F(*Mlu* I)	CGACGCGTCCATGCGGAGATTGCGGGGTTTT
Ib SAUR36-pNRT-R(*Sal* I)	GCGTCGACTCACCGCCGGCGGGAAACCATTCTG
Primers for qRT-PCR	
Ibactin-F	AGCAGCATGAAGATTAAGGTTGTAGCAC
Ibactin-R	TGGAAAATTAGAAGCACTTCCTGTGAAC
IbSAUR36-F	AAGGCCAAGGCTATTTGCTCTAA
IbSAUR36-R	CTGATGATCCGGGTCTGAACG
IbYUCCA6-F	TGCATAGCCTCTTTGTGGCA
IbYUCCA6-R	TTGTTCGGTCGGGTATGTCG
IbTAR2-F	GCTTGAAGTCTGGGCTCCAT
IbTAR2-R	TCCGGTCCCATTGACAACAG
IbUGT74-F	CTGCCATTGGGTGTTTCGTG
IbUGT74-R	CGCCGACCTTCCATATCTCC
IbAUX1-F	TAGGGATGCACGACACGAAG
IbAUX1-R	CGGGGATGATGTAGACGGTG
IbIAA26-F	TGAAGGTGGAAACCGCAACA
IbIAA26-R	TGCTTCCCACGGCTTAGAGT
IbJAZ-F	ACTTGCCAATTGCCAGGAGA
IbJAZ-R	GGCTTAGATGAACCCGCCAT
Ib4CL-F	CCATAGCTAAGAGCCCTGCC
Ib4CL-R	CTGACCAAGTTTGGCGTTGG
IbCAD-F	GGTTGCTGACGAGCACTTTG
IbCAD-R	ACAACGCCAATGTGCATTCC
IbNAC83-F	TCCTAATGGCAACAGGTCAA
IbNAC83-R	AAGAATATGCGGCACAACAC
